# Antioxidant Activity of γ-Oryzanol: A Complex Network of Interactions

**DOI:** 10.3390/ijms17081107

**Published:** 2016-08-09

**Authors:** Igor Otavio Minatel, Fabiane Valentini Francisqueti, Camila Renata Corrêa, Giuseppina Pace Pereira Lima

**Affiliations:** 1 Department of Chemistry and Biochemistry, Institute of Bioscience, Sao Paulo State University, Botucatu 18618-689, Brazil; igorminatel@hotmail.com; 2 Department of Pathology, Botucatu Medical School, Sao Paulo State University, Botucatu 18618-970, Brazil; fabiane_vf@yahoo.com.br (F.V.F.); ccorrea@fmb.unesp.br (C.R.C.)

**Keywords:** γ-oryzanol, ferulic acid, antioxidant capacity, dyslipidemia, obesity, inflammation

## Abstract

γ-oryzanol (Orz), a steryl ferulate extracted from rice bran layer, exerts a wide spectrum of biological activities. In addition to its antioxidant activity, Orz is often associated with cholesterol-lowering, anti-inflammatory, anti-cancer and anti-diabetic effects. In recent years, the usefulness of Orz has been studied for the treatment of metabolic diseases, as it acts to ameliorate insulin activity, cholesterol metabolism, and associated chronic inflammation. Previous studies have shown the direct action of Orz when downregulating the expression of genes that encode proteins related to adiposity (CCAAT/enhancer binding proteins (C/EBPs)), inflammatory responses (nuclear factor kappa-B (NF-κB)), and metabolic syndrome (peroxisome proliferator-activated receptors (PPARs)). It is likely that this wide range of beneficial activities results from a complex network of interactions and signals triggered, and/or inhibited by its antioxidant properties. This review focuses on the significance of Orz in metabolic disorders, which feature remarkable oxidative imbalance, such as impaired glucose metabolism, obesity, and inflammation.

## 1. Introduction

Grains are the most common staple food consumed worldwide. Taking into account that rice (*Oryza sativa* L.) constitutes the principal grain in the human diet, and since it feeds over half of the world’s population, is very important to consider its constituents, such as γ-oryzanol (Orz), fiber, γ-amino butyric acid, and vitamins. Therefore, the beneficial effects attributed to brown rice (BR) consumption must consider the synergic interaction of all these bioactive constituents. The consumption of BR (unpolished) in regular meals is strongly recommended, since the polishing step to obtain white rice reduces approximately 94% of the grain’s Orz content [1]. In addition, the polishing process removes other compounds that exert antioxidant activities, such as phenolics, tocopherols, and tocotrienols [1]. Furthermore, Orz composition and its amounts are variable among rice cultivars [2]; it is unequally distributed in the grain, with higher levels present in the bran layer and lower concentrations in the kernel. Strategies to improve BR’s bioactive components include affecting the germination process. By inducing germination of the whole rice grain, its compounds are remarkably increased [3], whereas the Orz content is slightly higher in germinated rice than in BR, and this is cultivar-dependent [4].

**Figure 1:**
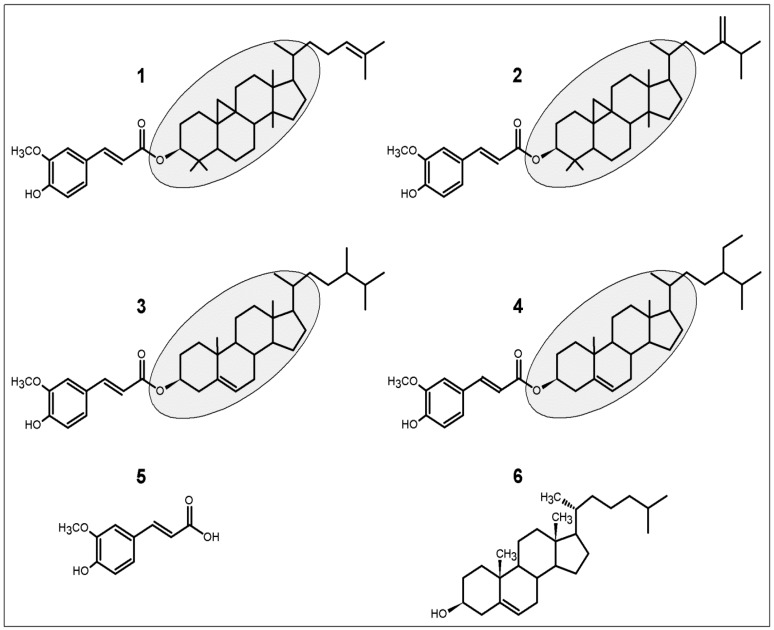
Molecular structures of the four main γ-oryzanol (Orz)’s components (**1**–**4**). Chemical structures are composed by ferulic acid and steryl ferulates (gray background). (**1**) cycloartenyl ferulate; (**2**) 24-methylenecycloartanyl ferulate; (**3**) campesteryl ferulate; and (**4**) sitosteryl ferulate. In the human body, Orz can be metabolized to (**5**) ferulic acid, and steryl ferulates closely similar to (**6**) cholesterol.

Orz comprises a mixture of ferulic acid (FA) esters and phytosterols (sterols and triterpenic alcohols) [5,6]. At least 10 steryl ferulates were identified in Orz, such as cycloartenyl ferulate, 24-methylenecycloartanyl ferulate, campestenyl ferulate, campesteryl ferulate, stigmastenyl ferulate, sitosteryl ferulate, ∆^7^-stigmastenyl ferulate, stigmasteryl ferulate, campestanyl ferulate, and sitostanyl ferulate [5]. Among these, cycloartenyl, 24-methylenecycloartanyl, campesteryl and sitosteryl ferulates predominate (Figure 1). The Orz constituents are commonly purified by high performance liquid chromatography (HPLC), whereas to identify isomers or the molecular variability of these constituents, methods as crystallization, nuclear magnetic resonance, and mass spectrometry (MS), have been employed [5,7,8,9]. However, the most suitable method to identify and quantify Orz with more sensibility is the liquid chromatography coupled to MS/MS [8,10]. The higher number of components identified by this method suggest that it is more recommended to identify and quantify Orz in biological tissues and fluids.

To better understand the mechanisms underlying the health benefits of Orz, and its interaction with different molecules, is necessary to consider the molecular structure of its metabolites. The consumption of Orz has been proved to be safe, with no relevant side effects reported. However, the most of the data available comes from studies in vitro or from animal models. In a recent study, Szcześniak et al. revised several of these studies and concluded that beneficial effects of Orz are due to its antioxidant activity and modifications in lipids metabolism [11]. A remarkable lack of specific dosages have used in animal models (doses range to 1–2000 mg/kg of body weight), or in vitro (doses range to 0.1–1000 µmol) [11]. Nevertheless, is still necessary to clarify the exact mechanisms of action and confirm the results obtained in human studies. For example, in mildly hypercholesterolemic men a daily dose of 50 mg of Orz, for 4 weeks, lowered total cholesterol, low-density lipoprotein (LDL) cholesterol, and LDL/high-density lipoprotein (HDL) cholesterol ratio by 6.3%, 10.5%, and 18.9%, respectively; whereas, increasing this dose to 800 mg/day did not enhance the pattern of lowering lipids [12].

Steryl ferulates arising from Orz share certain similarities with cholesterol (Figure 1). As an essential component of all mammalian cells, cholesterol is also an important structural component of myelin and a precursor of oxysterols, steroid hormones, and bile acids [13]. Orz has been shown to reduce plasma cholesterol levels and hepatic intake [14,15]; hence, it can affect different cell functions in the human organism. A great variety of biological effects has been attributed to Orz, such as antidiabetic [16], antioxidant [1,17], and anti-inflammatory activities [18]. However, until recently, the most studied Orz-related activities include its hypolipidemic and anti-obesity effects [15,16,19,20]. These potential health benefits are mainly verified through the introduction of a diet high in BR or germinated BR (GBR). Moreover, not only is Orz responsible for these effects, FA (the major metabolite of Orz) has been shown to improve lipid metabolism, hypertension, and glucose tolerance [15,21]. Here, we describe the wide range of beneficial activities arising from Orz’s antioxidant activity, and we then discuss how this compound is coupled to a number of health benefits.

## 2. Antioxidant Activity of γ-Oryzanol

In order to establish the beneficial effects of Orz in the antioxidant defense of cellular systems, it is important to consider that dietary antioxidants are essential for maintaining normal cellular functions and to ensure body homeostasis. Nevertheless, the regulation of a redox mechanism through dietary means is currently gaining considerable traction in the field of human and food sciences.

Oxidative stress results in a deleterious process that culminates in the damage of cell structures, including membranes and lipids, as well as proteins and DNA [22]. Reactive oxygen species (ROS) are constantly produced by enzymatic and non-enzymatic reactions. The major reactions catalyzed by enzymes that generate ROS include those involving NADPH oxidase, nitric oxide synthase (NOS), xanthine oxidase, arachidonic acid, and metabolic enzymes such as the cytochrome P450 enzymes, cyclooxygenase, and lipoxygenase. Non-enzymatic production of ROS comes from the mitochondrial respiratory chain. The major ROS produced in the human organism include singlet oxygen (^1^O_2_), superoxide anion (O_2_^•−^), hydroxyl radical (OH^•^), hydrogen peroxide (H_2_O_2_) and organic peroxides [23]. In addition, other molecules that affect oxidative balance are the reactive nitrogen species (RNS), such as nitric oxide (NO), nitrite (NO_2_^−^); carbon monoxide (CO); hydrogen sulfide (H_2_S) and its anion HS^−^ [23].

Oxidative imbalance is responsible for producing several reactive molecules, which are scavenged by Orz or its metabolites. The consumption of high-fat diets (HFD) has been shown to induce the formation of free radicals and ROS, resulting in lipid peroxidation and oxidative stress [24]. Orz and FA suppressed lipid peroxidation in mice fed a HFD [14] consumption based on a diet that included 15 mg/day of both compounds lowered plasma and erythrocyte thiobarbituric acid reactive substances (TBARS), when compared to control mice fed the HFD alone [14]. This finding illustrates that Orz and FA can act as ROS scavengers and prevent lipid peroxidation. Furthermore, the prevention of lipid peroxidation avoids lipotoxicity, which is associated with mitochondrial dysfunction, and formation of cellular ROS. In addition, these compounds have the capacity to reduce glucose-6-phosphate dehydrogenase (G6PD) [25], which promotes the expression of pro-oxidative enzymes NAPDH oxidase and NOS. Metabolites of Orz can induce different antioxidant responses in the organism, as observed in the serum levels of total antioxidant capacity (TAOC) and malondialdehyde (MDA) in rats [15]. In serum, reduced TAOC and increased MDA content was induced by HFD. However, FA treatment better improved TAOC and MDA levels when compared to Orz [15].

Regardless of the material (BR, bran, or isolated compounds) used to assess the antioxidant capacity of rice, it is controversial to strictly assign this potential to a given compound in isolate. Phenolic acids, tocopherols, tocotrienols, carotenoids and Orz are typical constituents of rice. Different rice cultivars may contain 8–10 times more Orz than vitamin E [2,17], which is considered one of the most effective antioxidants due to its biodisponibility. Nevertheless, the three major Orz metabolites (cycloartenyl, 24-methylenecycloartanyl and campesteryl ferulates) had higher antioxidant activities against cholesterol oxidation when compared to α- and γ-vitamin E isomers [17]. Among these, the highest antioxidant activity was imputed for 24-methylenecycloartanyl ferulate, which demonstrated variable antioxidant activity of the metabolites [17]. In another report, cycloartenyl, 24-methylenecycloartanyl, and β-sitosteryl ferulates, and FA showed a strong free radical scavenging and antioxidative protection of lipid peroxidation, which were comparable to α-tocopherol [26]. The hydroxyl group on the phenolic ring and an electron delocalization induced by ROS are important characteristics evolved in antioxidant activity of phytosterols [17,27]. In addition to the high amounts of Orz, its major metabolite ferulic acid presents the CH=CH–COOH group (cinnamic acid) that ensures an efficient antioxidant activity [27]. However, the major effect of Orz as an antioxidant is probably due to its capacity to prevent lipid peroxidation and the resulting oxidative stress. Thus, all the potential health benefits associated with Orz intake should be interpreted by considering its antioxidant capacity and other metabolic interactions.

Another important situation to consider when examining oxidative stress associated with redox imbalance is the resultant mitochondrial dysfunction. Different antioxidant systems are activated in cells, which fight the ROS produced and includes antioxidant molecules like superoxide dismutase (SOD), catalase [28], and glutathione [29,30]. SOD is responsible for catalyzing the dismutation of O_2_^−^ into H_2_O_2_, which is converted into H_2_O and O_2_ by catalase. Glutathione transferases comprise a super family of proteins that carries several redox regulations and occurs in all cellular life forms [31]. The fine regulation of these antioxidant systems is essential to prevent mitochondrial dysfunction, and its deregulation has long been implicated in the pathogenesis of Parkinson’s disease (PD) [32]. The production of free radicals and oxidative stress are among the deleterious factors associated with neuronal mitochondrial dysfunction. In a *Drosophila melanogaster* model of PD induced by rotenone, Orz improved antioxidant defenses, prevented oxidative stress, and attenuated mitochondrial dysfunction [33]. A significant increase in antioxidant enzymes (such as catalase, superoxide dismutase, and glutathione-S-transferase) was also observed and linked to abrogation of deleterious MDA and ROS produced by rotatone [33]. These activities are likely associated to the inhibition of free radical generation and the consequent prevention of inflammation progress.

## 3. Relation between γ-Oryzanol and Glucose Metabolism

At the cellular and molecular levels, oxidative stress is considered a key factor in the development of insulin resistance, impaired glucose, and diabetes. Many studies have indicated that BR ameliorates glucose metabolism [20,34,35,36]. Nevertheless, this statement is more accurate when one considers all the bioactive compounds present in rice. Mice fed a HFD plus BR showed ameliorated glucose tolerance and insulin resistance [35]; however, in the same study, a daily oral dose of Orz exerted the same effects as BR, suggesting that Orz or its metabolites are primarily responsible for the modulation of glucose metabolism [35].

**Figure 2:**
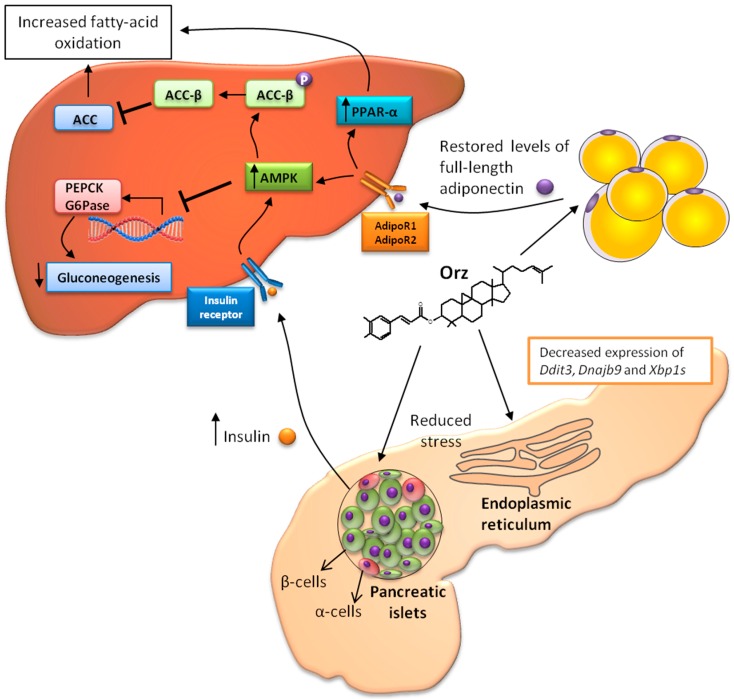
Synergic interaction of Orz with organs-organelles. Endoplasmic reticulum (ER) stress results in misfolded protein accumulation and leads to pancreatic β-cell death by apoptosis. Orz decreases the expression of ER stress-responsive genes *Ddit3*, *Dnajb9* and *Xbp1s*, and it consequently enhances β-cell insulin production. In addition, Orz improves the adipocyte production of adiponectin. Increased levels of insulin and adiponectin can activate 5′-AMP-activated protein kinase (AMPK) (via AdipoR1), which reduces the expression of phosphoenolpyruvate carboxykinase (PEPCK) and G6Pase, and inhibits gluconeogenesis. Furthermore, AMPK induces β-isoform of coenzyme A carboxylase (ACC-β) phosphorylation, which inhibits acetyl coenzyme A carboxylase (ACC) and results in increased fatty-acid oxidation. Full-length adiponectin activates peroxisome proliferator-activated receptors (PPAR-α) (via AdipoR2) and, thereby stimulating fatty-acid oxidation and decreasing triglyceride content in the tissues.

A close relationship exists between obesity and insulin regulation. Adiponectin produced by adipocytes has been shown to modulate glucose and lipid metabolism in insulin-sensitive tissues, such as liver and skeletal muscle [37]. Furthermore, obesity promotes adipocytes dysfunction and results in a consequently decreased level of adiponectin secretion [38]. In an stress-induced model of hypoadiponectinemia, Orz restored the globular and full-length adiponectin levels [39]. Full-length adiponectin is related to phosphorylation and activation of 5′-AMP-activated protein kinase (AMPK). AMPK phosphorylation positively regulates glucose metabolism and insulin sensitivity by reducing the expression levels of molecules involved in gluconeogenesis [40], such as phosphoenolpyruvate carboxykinase (PEPCK) and glucose-6-phosphatase (G6Pase) in hepatocytes [37]. In addition, the activation of AMPK results in the phosphorylation of the β-isoform of coenzyme A carboxylase (ACC-β), which inhibits acetyl coenzyme A carboxylase (ACC) [37], and consequently results in increased fatty acid oxidation (Figure 2).

Rodents fed a diet supplemented with Orz and FA were shown to exhibit regulated type 2 diabetes parameters, as their fasting glucose levels improved and their levels of glucose reduced during an oral tolerance test [15]. These effects may be explained by the results reported by Son et al., who showed that mice fed a HFD supplemented with either 0.5% Orz or 0.5% FA exhibited significantly lower blood glucose levels, and G6Pase and PEPCK activities, as well as higher glycogen and insulin concentrations, and glucokinase activity [25]. Insulin suppresses the expression of many hepatic genes associated with diabetes [41], including *G6Pase* and *PEPCK*. Both genes are essential for the regulation of hepatic gluconeogenesis, and its suppression represents an important step in type 2 diabetes control [42].

Another organ involved in the type 2 diabetes pathogenesis is the pancreas. Endoplasmic reticulum (ER) stress in pancreatic islet cells is linked to progressive β-cells dysfunction, apoptosis, and insulin resistance [43]. The ER is the cellular organelle in which protein synthesis, folding, and sorting take place. However, the development of stress in this organelle is accomplished by several metabolic disorders, resulting from misfolded protein accumulation and ROS formation. Although the organ-specific action of Orz in the pancreas is still unclear, following oral administration, Orz reaches its maximum plasma concentration in about 1 h. The distribution of Orz occurs mainly in the brain, whereas considerable amounts are found in the pancreas [44]. In the pancreas, Orz has been shown to decrease the expression of ER stress-responsive genes such as *Ddit3* (CCAAT/enhancer-binding protein-homologous protein), *Dnajb9* (ER resident DNAJ 4), and the spliced form of X box binding protein 1 (*Xbp1s*) [44]. These results suggest that Orz prevents ER stress-induce apoptosis, and it consequently enhances β-cell insulin production. Although Orz absorption by adipose tissues is lower than in the pancreas [44], it might improve adiponectin levels as discussed earlier. As Orz exerts its effects in the pancreas (it decreases ER stress and improves insulin secretion) and in adipose tissue (it improves adiponectin levels), it is feasible that Orz may display synergic effects in different organs, particularity as they relate to glucose metabolism (Figure 2). Improved levels of adiponectin and insulin culminates in liver AMPK activation. Adiponectin receptors 1 (AdipoR1) and 2 (AdipoR2) in the liver are stimulated by full-length adiponectin and they activate the phosphorylation of AMPK and PPAR-α, respectively [45]. Both pathways can increase fatty acid oxidation and lead to decreased triglyceride content [45,46]. AMPK stimulates fatty acid oxidation and ketogenesis, leading to reductions in cholesterol synthesis and lipogenesis. This kinase protects against lipid-induced hepatic disorders and consequently reduces ER stress [43].

## 4. Anti-Obesity Effects of γ-Oryzanol

The rising prevalence of overweight and obesity (body mass index ≥ 30 kg/m^2^) has become a global pandemic [47,48]. Improvements in quality of life and increases in income have created nutritional transitions and changes in dietary habits, such as the increased consumption of foods rich in fat and sugar, and of low nutritive quality. These changes are directly associated with obesity and the development of chronic diseases, such as type 2 diabetes mellitus (T2DM), cardiovascular diseases, dyslipidemia, and some cancers [49]. The consumption of whole grains, such as BR, is a promising approach to manage or prevent obesity and associated diseases. Most of these preventive effects are attributed to dietary fibers, since individuals with high intakes of dietary fiber face lower risk for developing obesity, moreover, the high consumption of fiber significantly contributes to weight loss [50].

The potential health benefits associated with the consumption of BR was evident in a study conducted in individuals with metabolic syndrome [16]. BR consumption resulted in decreased body weight, total cholesterol, and LDL-cholesterol levels, as well as lower postprandial concentrations of insulin and glucose, when compared to individuals consuming white rice [16]. However, dietary fibers from rice have either a slight (or no) effect on total cholesterol, triglycerides, and free fatty acids (FFA) blood levels [34,51]. Thus, these effects may be promoted by Orz and FA, as they showed a significant decrease in the body weight of rodents that were fed diets rich in fat and sugar [15,25,35]. The consumption of HFD is related to increased body weight gain and the development of local and systemic oxidative stress. This scenario constitutes an important triggering of metabolic syndrome and associated symptoms, such as hyperlipidemia, hyperglycemia, hypertension, insulin resistance, and hyperinsulinemia [52,53].

The steryl ferulates of Orz demonstrate antioxidant activity, as they donate hydrogen from their ferulic acid constituent [54]; beneficial effects may be reached by the antioxidant capacity of these bioactive compounds or by improving the metabolism of dietary components, such as cholesterol. Given the fact that FA is one of the major metabolites of Orz, it is possible assume that its effects in ameliorating obesity-related symptoms may be reached by Orz consumption. Wang et al. found that Orz and FA have similar effects in alleviating obesity and dyslipidemia in rats fed with HFD and high fructose diets. Both compounds were efficient in serum normalization of total cholesterol, triglycerides and LDL-cholesterol, and they induced FFA level reductions and high density lipoprotein (HDL) cholesterol increases [15]. Rong et al. showed that the addition of 1% of Orz to hypercholesterolemic diet for 7 weeks was able to decrease in 34% the plasma non-HDL-cholesterol of F1B Golden hamsters. In the same study, when Orz at 0.5% was added to hypercholesterolemic diet and animals fed for 10 weeks, a 57% reduction in plasma non-HDL-cholesterol was observed [55].

The capacity of Orz and FA to reduce triglyceride and cholesterol levels is directly induced by the suppression of hepatic lipogenesis, which occurs via regulation of the activities of NADPH-generating enzymes [14]. In addition, Orz and FA are able to improve the plasma and hepatic lipid profile by increasing faecal lipid excretion [14]. The available evidence suggests that intakes of 1.5–2.0 g of plant sterols may lower blood LDL-cholesterol by an average of 8.5%–10% [56,57]. In addition, the consumption of foods with low amounts of saturated fat and cholesterol, in association with the intake of sterols, can exacerbate LDL reduction by 20% [58].

It is noteworthy that Orz shares certain molecular similarities with cholesterol (Figure 1). Thus, it is pertinent to consider that phytosterols exert their cholesterol-lowering effects by decreasing cholesterol micellarization. Inside the intestinal lumen, dietary phytosterols are solubilized in micelles by bile acids, prior diffuse to enterocytes. Phytosterols have a higher solubility and affinity to the bile salt micelles than cholesterol and they may be effective for displacing cholesterol in micellarization [59]. In addition, phytosterol esters interact with digestive enzymes—particularly pancreatic cholesterol esterase (PCE). This enzyme is responsible for hydrolyzing lipids before micelle formation. In contrast to esters, free phytosterols show no effect on cholesterol ester hydrolysis [60]. The serum availability of FFA in the body is essential to induce hepatic lipogenesis, and Orz may control this mechanism by lowering FFA and reducing hepatic triglyceride synthesis [15]. This result is likely obtained by the decreased expression of liver X receptor α (LXRα), fatty acid synthase (FAS), and stearoyl coenzyme-A desaturase-1 (SCD-1) [15].

Unregulated appetite in humans is mainly derived from a leptin deficiency [61]; leptin is an adipocyte-derived hormone that acts on a subset of hypothalamic neurons to regulate food intake, thermogenesis, and the blood glucose levels [62]. Leptin inhibits food intake, stimulates cell energy expenditure, and results in a reduction of the body’s fat stores [62]. However, the contrasting effects of Orz, and their ability to decrease serum leptin levels or to attenuate one’s preference for HFD have been reported [15,35]. In addition to its critical role in appetite regulation, leptin resistance is associated with overnutrition and hypothalamus ER stress. Increased levels of FFA and overnutrition are conditions that lead to ER stress, and trigger a dysfunctional protein folding [61]. The intracellular accumulation of misfolded proteins results in ER stress and leads to the consequent activation of a complex network known as the unfolded protein response [63]. These mechanisms are directly involved in obesity, insulin resistance, and type 2 diabetes [63]. However, the reversing of ER stress and its associated improvement in the protein folding process resulted in increased insulin sensitivity and reverted type 2 diabetes in obese mice [64]. Kozuka et al. (2012), found that Orz attenuated the ER stress, improved glucose metabolism, and decreased plasma leptin. In addition, the authors observed an attenuated preference for dietary fat and the decreased expression of the following ER stress–responsive genes: CCAAT/enhancer-binding protein-homologous protein (*Chop*), endoplasmic reticulum resident DNAJ 4 (*ERdj4*), and the spliced form of X-box binding protein 1 (*Xbp1s*) [35].

**Figure 3:**
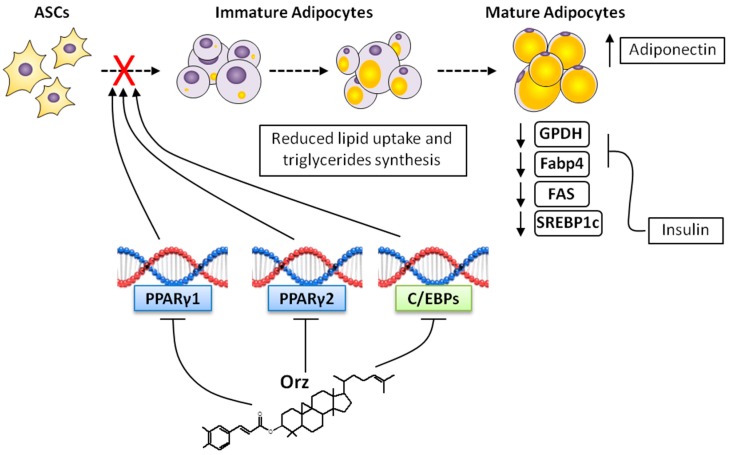
Steps of adipocytes differentiation and the possible effects of Orz. Adipose tissue stem cells (ASCs) are induced to differentiate into mature adipocytes through a complex network of signals. PPARγ and C/EBPα are the major regulators of this differentiation. By blocking PPARγ and C/EBPs expression, Orz exerts a direct influence on adipocytes differentiation. Immature adipocytes require lipid uptake, and Orz reduces the activities of glycerol-3-phosphate dehydrogenase (GPDH), fatty acid synthase (FAS), fatty acid binding protein 4 (Fabp4) and sterol regulatory element-binding protein-1c (SREBP-1c). This complex network is likely associated with systemic reduced insulin resistance, as well as to ameliorated ER stress and improved adiponectin secretion, as induced by Orz and its metabolites.

Adipogenesis is regulated by various transcription factors that coordinate innumerous protein responses and culminates into preadipocytes to adipocytes differentiation [65]. Among the transcription factors expressed in adipocytes, CCAAT-enhancer-binding proteins (C/EBPs) and PPARγ are key factors in this process, and their regulation may stop the complex transcriptional cascade and protein activation necessary for adipogenesis [66]. PPARγ is more essential for adipocyte differentiation than any other transcription factor [67]. Two isoforms of PPARγ (PPARγ1 and PPARγ2) are generated by the same gene; however, PPARγ1 is expressed in several tissues, whereas PPARγ2 expression is almost restricted to adipose tissue. Even in PPARγ2 knockout mice, an compensatory effect of PPARγ1 is observed [68]. In addition, the inhibition of PPARγ1 blocks adipocyte maturation (hypertrophy) and the expression of C/EBPα [69], suggesting that PPARγ is essential for adipocyte lipid accumulation (Figure 3). In addition to their direct effect on differentiation, PPARγ and C/EBPα can be stimulated by C/EBP-β and C/EBP-δ, factors that are expressed within the earlier phases of differentiation [65].

Additional factors that are present in parallel pathways are involved in PPAR-γ and C/EBPs regulation, such as sterol regulatory element-binding protein-1c (SREBP-1c). Furthermore, SREBP-1c is an important regulator of lipogenic enzymes such as ACC and FAS, and it controls the expression of PPARγ through the induction of endogenous ligand [70]. The insulin stimulation of 3T3-L1 preadipocytes significantly enhances SREBP-1c expression [70], whereas, ectopic expression of dominant-negative SREBP-1c has been shown to suppress differentiation through the regulation of genes involved in cholesterol homeostasis, fatty acid synthesis, and key enzymes involved in glycerolipid synthesis [71]. Ho et al. showed that extracts of GBR or BR down-regulated the expression of C/EBP-β, C/EBP-α, PPARγ, and SREBP-1c [19]. These data suggest that the bioactive compounds present in BR can control adipocytes differentiation. However, which compounds were present in the extract, and which accounted for anti-adipogenic effects, have not been defined. In contrast, Jung et al. showed that Orz induced 3T3-L1 cell differentiation into adipocytes by stimulating PPAR-γ and C/EBPα protein expression [72]. The results presented in the same study suggests that the differentiation of preadipocyte to adipocyte is induced by Orz and dependent of mammalian target of rapamycin complex 1 (mTORC1), which in turn can activate PPAR-γ [72]. The exactly participation of Orz in adipocyte differentiation has not been fully explained; however, this process seems to be strictly associated with ROS production [53], and an effective mechanism of action can be induced by the direct repression of transcription factors and/or ROS scavenging.

Important molecules involved in adipocytes maturation (hypertrophy) are glycerol-3-phosphate dehydrogenase (GPDH) [73] and fatty acid binding protein 4 (FABP4/aP2) [74]. Elevated GPDH in the adipose tissues is related to the increased synthesis of triacylglycerol [75]. However, the fatty acids used for glycerol 3-phosphate esterification must be derived from circulating lipoproteins and/or from food [75]. Extracts of GBR were associated with decreased GPDH activity in 3T3-L1 cells [19]. By blocking PPARγ and C/EBPs expression, or by decreasing GPDH activity, Orz is able to directly influence in lipid accumulation and fat mass expansion, which are characteristics of adipocyte hypertrophy (Figure 3). FABP4 is constantly released from the adipocytes and plays a crucial role in fatty acid uptake. Insulin secretion inhibits FABP4 release; it then coordinates lipid accumulation during adipocytes maturation [74]. FAS is a homodimeric enzyme responsible for the endogenous synthesis of fatty acids, which play a central role in the regulation of body weight and obesity [76]. In normal conditions, FAS converts excess carbohydrates into fatty acids, and they are then esterified to storage triacylglycerols. However, in situations of metabolic disorders as cancer [77] and obesity [76], FAS can be found in deregulated levels. Increased FAS expression in adipose tissue is linked with increased energy intake [76]. In 3T3-L1 cells being differentiated into adipocytes, both GBR and possibly Orz have shown interesting anti-adipogenic activities by decreasing the mRNA expression of *FAS* [19].

In the literature there are few articles that have investigated, in isolation, the anti-obesity effects of Orz or its metabolites. Nevertheless, the application of GBR and/or BR extract to assess this effect allows one to extrapolate some effects to Orz, particularly since this compound is certainly what differentiates between those health benefits that are attributed to rice and those not found in others grains.

## 5. γ-Oryzanol and Inflammation

**Figure 4:**
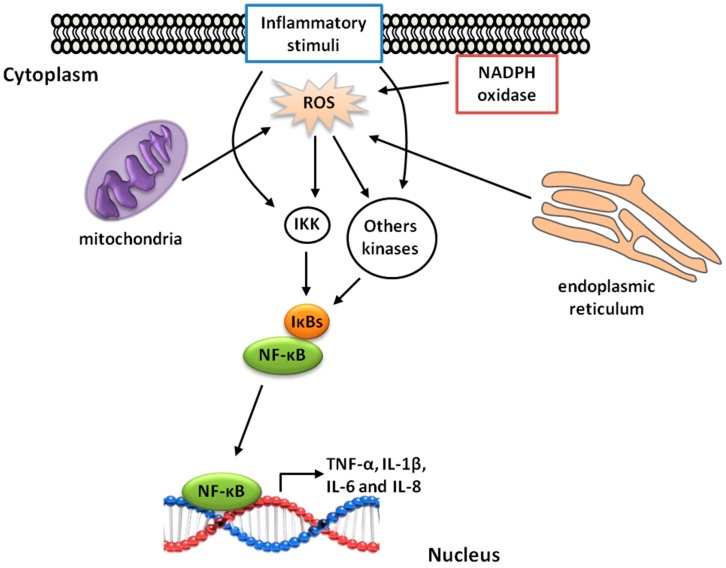
Signaling mechanisms of ROS-mediated nuclear factor kappa-B (NF-κB) activation. Inflammatory stimuli (proinflammatory cytokines, oxidative stress, etc.), and ROS produced by mitochondria, NADPH oxidase, and the endoplasmic reticulum triggers those kinase pathways that results in NF-κB activation. NF-κB can then translocate to the nucleus and induces target gene transcription, such as TNF-α, IL-1β, IL-6, and IL-8. Orz can reduce inflammation by scavenging ROS and consequently inhibiting the NF-κB pathways.

The Orz components may be useful to prevent the installation of inflammatory process in allergic reaction, since the non-polar structure of cycloartenyl ferulate proved to be capable of sequestering the immunoglobulin E and inhibit the allergic reaction mediated by mast cell degranulation [78]. There are several health benefits attributed to Orz due to its anti-inflammatory and antioxidant activities. The presence of inflammation increases ROS production inside the cell, either through NADPH oxidase or the mitochondrial electron transport chain [79]. These reactive molecules are directly related to the progression of inflammatory processes, as they induce cell injury and/or lead to the activation of redox-sensitive transcription factors (Figure 4). Some ROS arising from the plasma or organelles membrane can influence transcription by regulating the phosphorylation of transcription factors, whereas ROS arising from the perinuclear mitochondria or from a nuclear flavoenzyme can participate in transcriptional control by directly targeting DNA [23]. In addition, inflammation can be exacerbated by an ER folding process (which produces ROS), or by a disruption in this process, leading to unfold protein release and cell damage [80].

Among the transcription factors, nuclear factor-kappa B (NF-κB) is involved in the regulation of proinflammatory genes, which represents a key step in the production of proinflammatory cytokines such as tumor necrosis factor-α (TNF-α), IL-1β, IL-6, and IL-8 [81]. The role of Orz in regulating these cytokines was verified in an experimental model of colitis. Significant reductions in the mRNA expression of TNF-α, IL-1β, IL-6, and cyclooxigenase-2 were observed in mice treated with Orz. The same author described a reduction in the tissue infiltration of inflammatory cells [18].

NF-κB is a member of the Rel family of proteins that can form homodimers or heterodimers. The activity of NF-κB is regulated by inhibitory IκB proteins [81]. Inflammatory or redox cell stimulation induces the IκB kinase (IKK) pathway and results in a cascade of activations, such as MAP kinase, c-Jun amino-terminal kinases (JNK), and TNF receptor associated factor 1 (TRAF1) and 2 (TRAF2) [82]. NF-κB is trapped in the cytoplasm in stimulated cells and it is translocated into the nucleus following the presence of stimuli that include oxidative stress [82]. Interestingly, Islam et al. (2009) reported that phytosteryl ferulates of Orz were able to inhibit the nuclear translocation of NF-κB in LPS-stimulated RAW 264.7 macrophages. The exact mechanism that allows Orz or its metabolites to inhibit NF-κB activity remains unclear; however, this inhibition appears to be induced by scavenging ROS or blocking molecules that active transcription factors such as TNF-α, IL-1β, IL-6, and cyclooxigenase-2 (Figure 4).

## 6. Conclusions

Orz plays an important role, at least in part, in preventing some lifestyle diseases related to oxidative stress and high fat intake. Before attributing some organ-specific effects to Orz, it is necessary to consider its variable uptake by cells, as well as its interactions with the transcription factors, ROS, and proteins/enzymes involved in specific health disorders. Orz’s radical scavenging capacity triggers a complex network of interactions and culminates in organ-cell specific responses. These interactions are related to reducing the processes involved in ER stress, lipid intake, and peroxidation, as well as those related to improved glucose metabolism. Orz has attracted attention as a functional food with several beneficial interactions in organism, particularly given its antioxidant, anti-obesity, and anti-inflammatory properties, as well as its ability to improve insulin resistance and hepatic metabolism. All of these disorders exhibit a characteristic redox imbalance. However, it is certainly clear that much more research is required to recognize how Orz and its metabolites are required (and used) by organs or cells when they occur in association.
